# Time Course of a Single, 0.6 mg/kg Dose of Rocuronium Neuromuscular Block During Sevoflurane or Propofol Anesthesia in Infants—A Prospective, Randomized Trial

**DOI:** 10.3390/jcm14186459

**Published:** 2025-09-13

**Authors:** Béla Fülesdi, Péter Luterán, Mena Boktor, László Asztalos, György Nagy, Sorin J. Brull, Csilla Molnár

**Affiliations:** 1 Department of Anesthesiology and Intensive Care, Faculty of Medicine, University of Debrecen, Nagyerdei krt. 98, 4030 Debrecen, Hungary; luteranpeti@gmail.com (P.L.); mena.boktor45@gmail.com (M.B.); asztaloslasz@gmail.com (L.A.); gynagy1986@gmail.com (G.N.); csmolnar@med.unideb.hu (C.M.); 2 Edömér Tassonyi Neuromuscular Research Group, 4032 Debrecen, Hungary; sjbrull@me.com; 3 Department of Anesthesiology and Perioperative Medicine, Mayo Clinic College of Medicine, 4500 San Pablo Rd., Jacksonville, FL 32066, USA

**Keywords:** quantitative neuromuscular monitoring, rocuronium, infants, pediatric neurosurgery, sevoflurane, propofol

## Abstract

**Background:** There is little data available in infants on the extent to which inhalational anesthetics prolong the effects of neuromuscular blocking agents compared with intravenous agents. Here, we assessed the differences between the neuromuscular blocking effects (duration and recovery time) of a single dose of rocuronium during propofol vs. sevoflurane anesthesia. **Methods:** The prospective study enrolled 20 infants (4–12 months of age) scheduled for craniosynostosis surgery, randomly assigned to receive general anesthesia maintenance with sevoflurane or propofol. All patients received 0.6 mg/kg rocuronium as a single bolus dose to facilitate tracheal intubation and surgery. Primary study endpoint was the clinical duration of rocuronium, from administration until spontaneous recovery to a train-of-four ratio (TOFR) > 0.90. Secondary endpoints were times for reappearance of the first, second, third, and fourth twitches of the TOF (T1, T2, T3 and T4, respectively) in the two patient groups. **Results:** There were no differences in the infants’ age (sevoflurane maintenance: 5.8 ± 2.4 months; propofol maintenance: 6.7 ± 3.1 months, *p* = 0.47) or weight (sevoflurane: 7722 ± 1644 g; propofol: 7433 ± 1782 g, *p* = 0.71). Rocuronium onset time was 101.0 ± 55.0 s in the sevoflurane group and 83.4 ± 47.9 s in the propofol group (*p* = 0.46). Total duration of anesthesia was comparable in the sevoflurane (122.0 ± 23.8 min) and propofol (107.7 ± 25.2 min, *p* = 0.18) groups. Rocuronium recovery to TOFR > 0.9 required 136 min (CI: 123.7–149.5 min) in the sevoflurane group and 61.5 min (CI: 58.0–101.0 min) in the propofol group (*p* < 0.001). **Conclusions:** In infants, sevoflurane maintenance enhances the neuromuscular blocking effect of a single, 0.6 mg/kg BW dose of rocuronium as compared to propofol maintenance. After discontinuation of sevoflurane, additional time is necessary to reach the acceptable TOFR >0.9 needed before tracheal extubation. The present study further underscores the importance of objective (quantitative) neuromuscular monitoring in infants to guide intraoperative management and prevent residual neuromuscular block.

## 1. Introduction

As shown in a recent meta-analysis, avoidance of the use of neuromuscular blocking agents for safe tracheal intubation is not recommended in the pediatric population because of the higher risk of failed intubations, airway injury, and respiratory adverse events [1]. This finding is particularly true in children under 24 months of age, in whom the frequency of respiratory adverse events can reach 20% [2]. Recent adult guidelines propose the use of objective neuromuscular monitors when non-depolarizing neuromuscular blocking agents are administered [3,4], although clinical guidelines for management of neuromuscular block in pediatrics are currently under development.

Neuromuscular monitoring and the diagnosis of residual block in the pediatric population are of paramount importance, as clinical signs of residual block in this population are even harder to diagnose due to communication difficulties. Additionally, the oxygen reserve in children is lower, and they may be more vulnerable to airway collapse. It is reported that the incidence of residual neuromuscular block in the pediatric population may be as high as 28% [5]. A recent survey by the Society for Pediatric Anesthesia found that only 40% of pediatric anesthesiologists regularly assess neuromuscular function during and after surgery, and the use of neuromuscular monitors is inversely correlated with years of practice [6].

From a clinical perspective, it is also important to emphasize that different anesthetics used for maintenance of anesthesia may have different effects on the duration and recovery of neuromuscular block; volatile agents enhance neuromuscular block both in adult [7,8] and pediatric [9] populations. Based on previous clinical reports, sevoflurane and isoflurane were the agents with the greatest potentiating effect of non-depolarizing neuromuscular blocking agents [7,9]. It is also known that the duration of neuromuscular block and recovery may be longer in infants than in children [10]. Data indicate that maturation of the neuromuscular junction may be an ongoing process in infants and thus the effect of the neuromuscular blocking agents and the interaction with maintenance anesthetics may be unpredictable [11,12]. Clinical studies on the pharmacokinetics of neuromuscular blocking agents in the pediatric population, and especially in infants, are scarce. Additionally, there is little data available on the extent to which inhalational anesthetics (e.g., sevoflurane) prolong the effects of neuromuscular blocking agents in this age group compared with intravenous agents (e.g., propofol). In the present study, we assessed the differences between the neuromuscular blocking effect of a single dose of rocuronium during propofol vs. sevoflurane maintenance of anesthesia.

## 2. Methods

This is a prospectively randomized study performed in infants aged 4–12 months who were scheduled for elective craniosynostosis surgery at the Department of Neurosurgery, University of Debrecen, Hungary. The protocol of the study was approved by the local (Medical Ethics Committee of the University of Debrecen, DE RKEB/IKEB 6644-2023, approval date: 13 December 2023) and national (Medical Research Council Ethics Committee for Clinical Pharmacology, registration number: BM/21031-0/2024 EKL, approval date: 21 August 2024) authorities. The study was registered prior to patient inclusion at the Clinical Trials registry EUCT number: 2024-515545-41-00; registration date: 3 September 2024, url: https://euclinicaltrials.eu/search-for-clinical-trials/?lang=en&EUCT=2024-515545-41-00 (accessed on 24 July 2025). Recruitment and inclusion of patients started on 6 September 2024. This manuscript adheres to the applicable CONSORT guidelines. In all cases, the study was explained to the parents in detail, who gave written informed consent to the study.

Inclusion criteria were infants undergoing elective craniosynostosis surgery. Exclusion criteria were diseases affecting neuromuscular function, severe liver and/or kidney failure, lack of parental consent, and known allergy to any of the medications.

In the original protocol, we intended to assess the effect of sevoflurane and propofol maintenance of anesthesia on the neuromuscular recovery using two different doses of rocuronium. In the first, non-randomized part, clinical anesthesiologists made a subjective decision whether they expected any difficulties with the intubation process. The clinical signs on which this decision was based were high or narrow palatal arches, facial distortion, short neck, increased neck circumference, and limited neck mobility. It was planned that in cases where a difficult airway was assumed, rocuronium 0.9 mg/kg body weight (BW) would be given, while in all other cases, a 0.6 mg/kg BW rocuronium would be given for tracheal intubation. Accordingly, the first power analysis considered the results of a previous study [13], which described that after a dose of 0.6 mg/kg BW, the muscle relaxant recovery time was 21 ± 4 min, while after a dose of 0.9 mg/kg BW, recovery time was 34 ± 11 min. Since the inhalation anesthetic sevoflurane prolongs recovery times to the same extent, 3 patients per rocuronium dose group (0.6 vs. 0.9 mg/kg) would be required for statistical significance.

**Figure 1:**
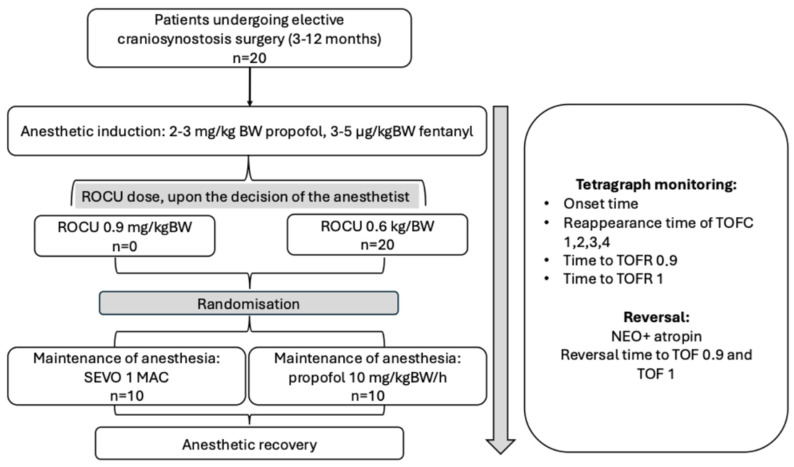
Flowchart (structured summary) of the study. ROCU indicates rocuronium; BW indicates body weight; MAC indicates minimum alveolar concentration; TOFC indicates train-of-four count; TOFR indicates train-of-four ratio; SEVO indicates sevoflurane.

For testing the second and main study question, we also performed a power analysis. We used the results from a previous randomized study [8], which found that the time needed for recovery to a train-of-four (TOF) ratio (TOFR) of 0.8 was 103 ± 30.7 min during sevoflurane maintenance, and 62 ± 21.1 min during propofol maintenance. Using an alpha of 0.05 and a power of 85%, 10 patients per group (total n = 20 patients) were necessary to determine whether sevoflurane, as compared to propofol, has an influence on the duration of reversal of neuromuscular block after a single 0.6 mg/kg BW dose of rocuronium. Thus, we included 20 patients and randomly allocated them to sevoflurane (inhalation agent) or propofol (intravenous agent) maintenance groups. Pre-sealed opaque envelopes were used for randomization; they were opened by the research anesthesiologist, who was also responsible for recording the neuromuscular monitoring results. Patient inclusion and allocation are shown in Figure 1.

The predefined primary endpoint was to compare the time needed to reach TOFR = 0.9 after a single, 0.6 mg/kg dose of rocuronium in the propofol vs. sevoflurane maintenance anesthesia groups. Secondary endpoints were times for reappearance of the first, second, third, and fourth twitches of the TOFR (T1, T2, T3 and T4, respectively) in the two patient groups. Additional endpoints were the necessity of reversal agent administration in the two groups.

### 2.1. Anesthesia Protocol

Anesthesia was induced in both groups using 2–3 mg/kg propofol and 3–5 μg/kg of fentanyl. Once the eyelash reflex was lost, 0.6 mg/kg rocuronium was injected intravenously as a bolus. For maintenance of anesthesia, propofol 10 mg/kg/h plus fentanyl 2–4 μg/kg/h were administered in the propofol (PROP) group, while 2.5% sevoflurane (end tidal concentration) corresponding to 1 minimum alveolar concentration (MAC) in 100% percent oxygen plus 2–4 μg/kg/h fentanyl were used in the sevoflurane (SEVO) group. Heart rate, non-invasive blood pressure, and core body temperature were also documented. The inspired and end-tidal concentrations of O_2_, carbon dioxide (CO_2_), and sevoflurane MAC were monitored by a multi-gas monitor incorporated in the Draeger Zeus (Draeger, Lübeck, Germany) anesthesia workstation.

### 2.2. Monitoring Neuromuscular Function

**Figure 2:**
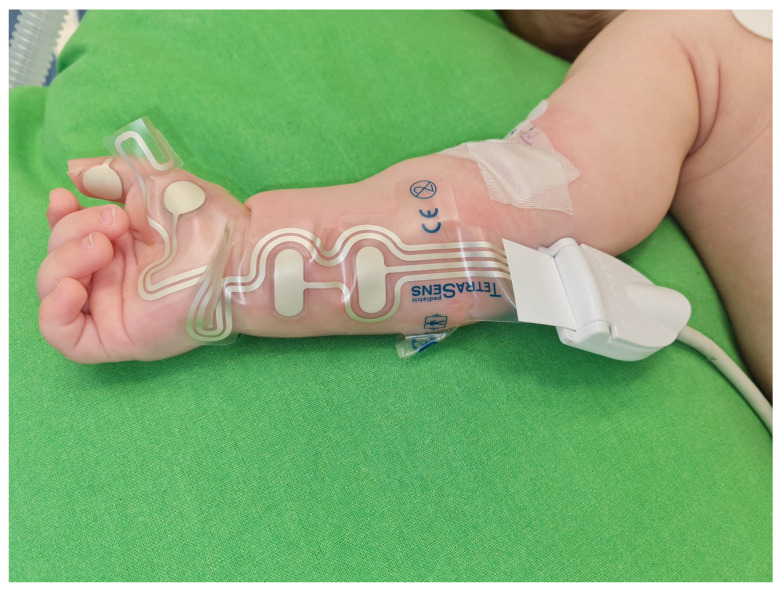
Placement of the pediatric TetraSens electrode.

A TetraGraph electromyographic (EMG) neuromuscular monitor (Senzime AB, Uppsala, Sweden) was used for monitoring neuromuscular function. We used the TetraSens Pediatric electrodes (Senzime AB), which are indicated in the pediatric population. The strip electrode was placed on the right volar aspect of the forearm with the proximal stimulating electrodes over the ulnar nerve and the distal sensing electrodes over the adductor pollicis muscle (Figure 2).

After the induction of anesthesia but prior to administration of neuromuscular blocking agents, the monitor underwent a self-calibration sequence (over 10–15 s) to determine the supramaximal stimulation current (mA) and ensure consistent stimulation over time. Then, a baseline TOFR was recorded. Thereafter, the auto-mode of the TetraGraph device was used for recording neuromuscular responses.

### 2.3. Statistical Analysis

All values were assessed by a normality test. Parameters with normal distribution are reported as means and standard deviation, whereas non-normally distributed data are reported as medians and 25–75% interquartile ranges. Depending on the distribution of the data, the appropriate t-tests or Mann–Whitney U-tests were used for statistical comparison. A *p* < 0.05 defined statistical significance. For better visualization of results, TOFC1–4 values are depicted in the graph as means and standard deviations.

## 3. Results

Twenty infants aged 4–12 months who underwent craniosynostosis surgery were enrolled. After randomized patient allocation to the SEVO and PROP maintenance groups, there were no differences in the infants’ age (SEVO: 5.8 ± 2.4 months; PROP: 6.7 ± 3.1 months, *p* = 0.47). No significant differences were detected between the weight of infants in the two groups (SEVO: 7722 ± 1644 g; PROP: 7433 ± 1782 g, *p* = 0.71).

The first study question of the original protocol could not be tested, because clinical anesthesiologists used a 0.6 mg/kg BW rocuronium dose in all cases, and no patients with a dose of 0.9 mg/kg BW rocuronium were included. After administration of 0.6 mg/kg BW rocuronium, intubating conditions were excellent in all cases, and endotracheal intubation was accomplished on the first attempt. Total duration of anesthesia was comparable in the two groups (SEVO: 122.0 ± 23.8 min, PROP: 107.7 ± 25.2 min, *p* = 0.18). Similarly, there were no differences in the duration of surgery between groups (SEVO: 64.1 ± 22.5 min, PROP: 64.2 ± 38.2 min, *p* = 0.92).

### 3.1. Results of Neuromuscular Monitoring

Onset time of rocuronium was 101.0 ± 55.0 s in the SEVO group and 83.4 ± 47.9 s in the PROP group (*p* = 0.46). Anesthesia induction was accomplished with the same drug combination (propofol and fentanyl) in both groups; propofol total doses were 22.9 ± 5.3 mg and 23.2 ± 4.9 mg, while fentanyl total doses were 29.7 ± 7.1 μg and 30.9 ± 6.6 μg in the SEVO and PROP groups, respectively.

Time course of neuromuscular block after administration of a single dose of 0.6 mg/kg BW rocuronium

Recovery times until train-of-four count of 1 (TOFC1) in the SEVO group (median: 69 min; CI: 39.7–109 min) were longer than those in the PROP group (median: 30 min; CI: 22–59 min), but the difference did not reach statistical significance (*p* = 0.1). Train-of-four count of 2 (TOFC2) recovery required a median of 81 min (CI: 53.2–119.5 min) in the SEVO group and 32.5 min (CI: 29–72 min) in the PROP group (*p* = 0.03). Train-of-four count of 3 (TOFC3) recovery required a median of 95 min (CI: 63–122.2 min) in the SEVO group, and 35.5 min (CI: 33–83 min) in the PROP group (*p* = 0.025). Train-of-four count of 4 (TOFC4) recovery required a median of 105 (CI: 69.2–127.5 min) in the SEVO group, and 50 (34–83 min) in the PROP group (*p* = 0.02).

**Figure 3:**
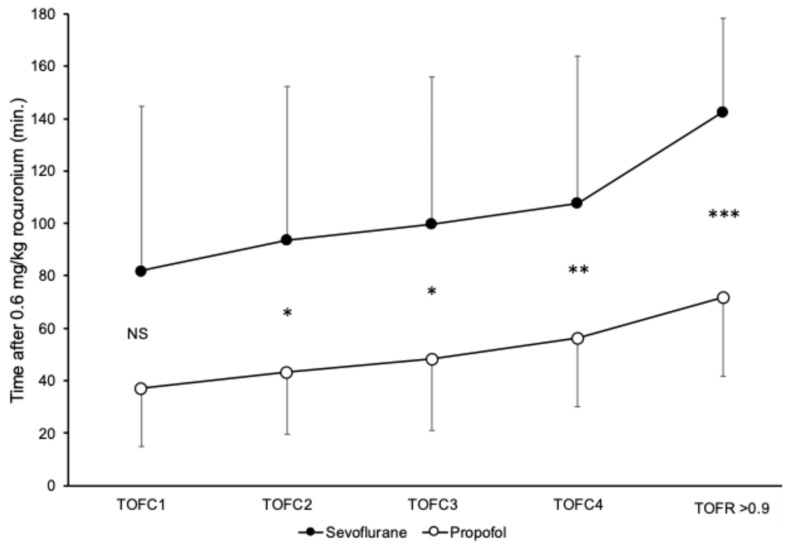
Results of quantitative neuromuscular monitoring after a single dose of 0.6 mg/kg BW rocuronium during sevoflurane and propofol maintenance. Means and standard deviations are presented. NS indicates non-significant * indicates *p* < 0.05; ** indicates *p* < 0.01; *** indicates *p* < 0.001 difference between groups. TOFC: train-of-four count; TOFR: train-of-four ratio.

Recovery to TOFR > 0.9 during administration of the maintenance drug required 136 min (CI: 123.7–149.5 min) after the single dose of rocuronium in the SEVO group and 61.5 min (CI: 58.0–101.0 min) in the PROP group (*p* < 0.001). Figure 3 provides a visual representation of the results, including time required for recovery of TOFC1–4 in both SEVO and PROP groups.

### 3.2. Comparison of Time Elapsed from Discontinuation of Maintenance Anesthetic and Return to TOFR 0.9

**Figure 4:**
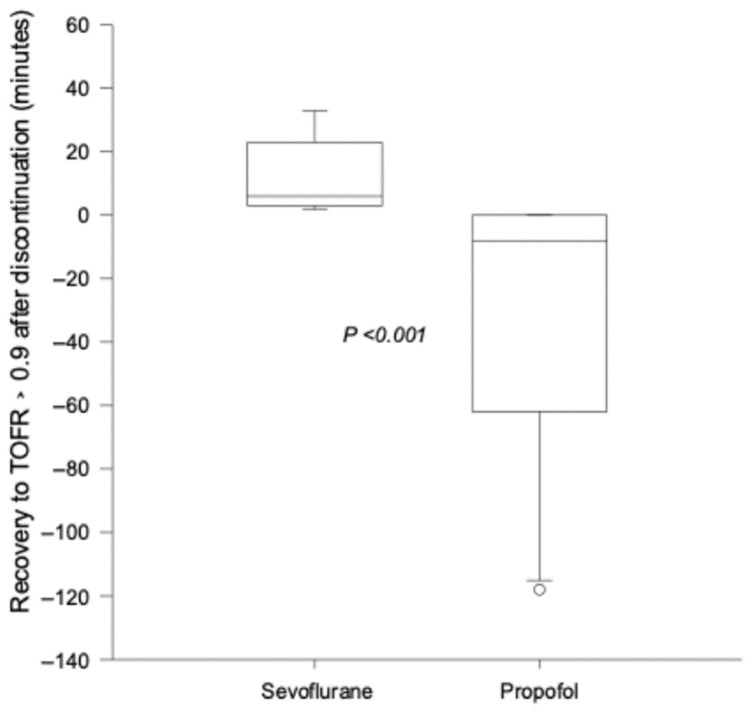
Time (in minutes) from the discontinuation of the maintenance anesthetic in the sevoflurane and propofol groups. TOFR = train-of-four ratio.

At the end of the surgical intervention, discontinuation of the sevoflurane anesthesia necessitated a 6 min (CI: 3.5–19 min) period to reach TOFR > 0.9. In the PROP group, TOFR > 0.9 was reached at the time of discontinuation of propofol in 3 cases, and in the other 7 cases, the TOFR > 0.9 was reached 2–118 min (median: 15 min) before propofol discontinuation. Figure 4 illustrates the differences in time between discontinuation of the maintenance drug and reaching TOFR > 0.9.

## 4. Discussion

As reported previously in children and adults, we found that sevoflurane maintenance enhances the neuromuscular blocking effect of a single, 0.6 mg/kg BW dose of rocuronium in infants as compared to propofol maintenance. This represents new information on the potentiation of rocuronium neuromuscular block induced by sevoflurane in this age group.

The initial goal of the original protocol could not be addressed in the present study, since no patients with difficult-appearing airway (necessitating a dose of 0.9 mg/kg BW) were enrolled. Our results support previous reports [14] that described similar intubating conditions in infants when using 0.45 mg/kg BW and 0.6 mg/kg BW of rocuronium, suggesting that our predetermined lower (0.6 mg/kg BW) dose of rocuronium likely would also have been sufficient in difficult airway patients.

In both groups, the induction of anesthesia occurred through the administration of a combination of propofol and fentanyl using the same doses. This was performed intentionally because we wanted to assess the effect of sevoflurane vs. propofol as a maintenance technique on the neuromuscular block characteristics in infants. The onset time of the single dose of 0.6 mg/kg rocuronium was 92.2 ± 51 s in our entire 20-patient cohort. This onset time is comparable with that reported in adults [8] and in children [15], but is faster than that reported in children aged 3–7 years [13]. It is known from previous pharmacokinetic studies that infants may need lower effective plasma concentrations for a given neuromuscular block effect because of the infants’ reported lower quantal content of acetylcholine at the neuromuscular endplate [16], which may explain these differences.

The main finding of the present study is that sevoflurane, as a maintenance drug, potentiates the effect of rocuronium in infants, as compared to propofol. Although the time to return of TOFC1 was longer in the SEVO group as compared with the PROP group, this difference did not reach statistical significance—although a difference between 61 and 30 min is clinically relevant. In the later phases of neuromuscular recovery, the return of TOFC2–4 responses in the sevoflurane group required, on average, almost twice as long as those in the sevoflurane group. The most marked difference between the recovery times was observed in the return to TOFR > 0.9. Sevoflurane prolonged the time of recovery to TOFR 0.9 by a median time of 74.5 min compared to propofol. The potentiating effect of the inhalational agents on the neuromuscular blocking effect of rocuronium is well-known [7,8,9]. In the adult population, a 103 min time to return to TOFR > 0.8 during sevoflurane maintenance, and a 62 min recovery time during propofol maintenance, were reported [8]. Although data on the spontaneous recovery times after a single bolus dose of rocuronium in infants during sevoflurane maintenance are lacking, a prolonged spontaneous recovery time was reported in children during sevoflurane maintenance compared to nitrous oxide-fentanyl anesthesia after administration of a rocuronium infusion [17]. Similarly to our study, sevoflurane as a maintenance agent delayed both the return of T1 and T4 responses to baseline. Sevoflurane may thus enhance the presynaptic effect of the non-depolarizing muscle relaxants during tetanic and train-of-four stimulation. Additionally, in infants, previous studies described development of significant fade in response to increasing stimulation frequencies and post-tetanic exhaustion after tetanic stimulation [18]. In healthy infants, post-tetanic facilitation may be lacking, and compound muscle action potential (cMAP) decreases can be observed, suggesting an incomplete maturation process of the neuromuscular junction [19]. Consequently, lower doses of neuromuscular blocking agents may be sufficient in infants, and the potentiating effect of maintenance drugs may not be predicted by simple extrapolation of data obtained in children.

As shown in Figure 4, TOFR > 0.9 in the propofol group was reached before the end of surgery and prior to emergence from anesthesia in a majority of infants. In the sevoflurane maintenance group, discontinuation of sevoflurane resulted in a fast recovery of the neuromuscular response to TOFR > 0.9. Similarly to our observations, other investigators reported a prompt return of the neuromuscular function in children [17].

Our report has certain limitations. The first part of the original protocol could not be performed because there was no clinical indication for the use of rocuronium 0.9 mg/kg dose, as originally planned. Hence, we randomized patients according to the second study aim. Second, this is a single-center, randomized study. Although the number of included patients met the requirements according to the power analysis, the results may not be generalizable, especially because of the high variability in the neuromuscular responses to neuromuscular blocking agents in the infant population. We also only administered a single dose of rocuronium and avoided administration of additional top-up (or continuous infusion) doses of neuromuscular blocking agents. However, our surgical data indicate that a single dose of 0.6 mg/kg BW rocuronium was sufficient for the entire surgical intervention in the majority of the cases; furthermore, we took advantage of the potentiating properties of sevoflurane and propofol to depress pharyngeal and laryngeal reflexes at the time of tracheal intubation and reduce spontaneous limb movements; thus, we did not consider it necessary to use an additional neuromuscular blocking agent dose before the end of the surgery.

## 5. Conclusions

In conclusion, in the present prospective randomized study, we show that sevoflurane as a maintenance anesthetic potentiates the neuromuscular blocking effect of a single dose of rocuronium in infants. We also show that after discontinuation of sevoflurane, rocuronium’s neuromuscular blocking effect may persist, and additional time is necessary to reach the acceptable TOFR > 0.9 needed prior to tracheal extubation. The present study underscores the importance of objective (quantitative) neuromuscular monitoring in infants to guide intraoperative management and prevent residual neuromuscular block.

## Data Availability

Data will be available from the corresponding author upon a reasonable request.

## References

[1] Disma N., Asai T., Cools E., Cronin A., Engelhardt T., Fiadjoe J., Fuchs A., Garcia-Marcinkiewicz A., Habre W., Heath C. (2024). Airway management in neonates and infants: European Society of Anaesthesiology and Intensive Care and British Journal of Anaesthesia joint guidelines. Eur. J. Anaesthesiol..

[2] Vanlinthout L.E., Geniets B., Driessen J.J., Saldien V., Lapré R., Berghmans J., Uwimpuhwe G., Hens N. (2020). Neuromuscular-blocking agents for tracheal intubation in pediatric patients (0–12 years): A systematic review and meta-analysis. Paediatr. Anaesth..

[3] Thilen S.R., Weigel W.A., Todd M.M., Dutton R.P., Lien C.A., Grant S.A., Szokol J.W., Eriksson L.I., Yaster M., Grant M.D. (2023). 2023 American Society of Anesthesiologists Practice Guidelines for Monitoring and Antagonism of Neuromuscular Blockade: A Report by the American Society of Anesthesiologists Task Force on Neuromuscular Blockade. Anesthesiology.

[4] Fuchs-Buder T., Romero C.S., Lewald H., Lamperti M., Afshari A., Hristovska A.-M., Schmartz D., Hinkelbein J., Longrois D., Popp M. (2023). Peri-operative management of neuromuscular blockade: A guideline from the European Society of Anaesthesiology and Intensive Care. Eur. J. Anaesthesiol..

[5] Scheffenbichler F.T., Rudolph M.I., Friedrich S., Althoff F.C., Xu X., Spicer A.C., Patrocínio M., Ng P.Y., Deng H., Anderson T.A. (2020). Effects of high neuromuscular blocking agent dose on post-operative respiratory complications in infants and children. Acta Anaesthesiol. Scand..

[6] Faulk D.J., Austin T.M., Thomas J.J., Strupp K., Macrae A.W., Yaster M. (2021). A Survey of the Society for Pediatric Anesthesia on the Use, Monitoring, and Antagonism of Neuromuscular Blockade. Anesth. Analg..

[7] Oris B., Crul J.F., Vandermeersch E., Van Aken H., Van Egmond J., Sabbe M.B. (1993). Muscle paralysis by rocuronium during halothane, enflurane, isoflurane, and total intravenous anesthesia. Anesth. Analg..

[8] Lowry D.W., Mirakhur R.K., McCarthy G.J., Carroll M.T., McCourt K.C. (1998). Neuromuscular effects of rocuronium during sevoflurane, isoflurane, and intravenous anesthesia. Anesth Analg..

[9] Woloszczuk-Gebicka B., Lapczynski T., Wierzejski W. (2001). The influence of halothane, isoflurane and sevoflurane on rocuronium infusion in children. Acta Anaesthesiol. Scand..

[10] Woelfel S.K., Brandom B.W., McGowan F.X., Cook D.R. (1993). Clinical pharmacology of mivacurium in pediatric patients less than off years old during nitrous oxide-halothane anesthesia. Anesth. Analg..

[11] Meretoja O.A. (1990). Neuromuscular blocking agents in paediatric patients: Influence of age on the response. Anaesth. Intensive Care.

[12] Goudsouzian N.G. (1980). Maturation of neuromuscular transmission in the infant. Br. J. Anaesth..

[13] Fuchs-Buder T., Tassonyi E. (1996). Intubating conditions and time course of rocuronium-induced neuromuscular block in children. Br. J. Anaesth..

[14] Rapp H.J., Altenmueller C.A., Waschke C. (2004). Neuromuscular recovery following rocuronium bromide single dose in infants. Paediatr. Anaesth..

[15] Scheiber G., Ribeiro F.C., Marichal A., Bredendiek M., Renzing K. (1996). Intubating conditions and onset of action after rocuronium, vecuronium, and atracurium in young children. Anesth Analg..

[16] Wierda J.M., Meretoja O.A., Taivainen T., Proost J.H. (1997). Pharmacokinetics and pharmacokinetic-dynamic modelling of rocuronium in infants and children. Br. J. Anaesth..

[17] Woloszczuk-Gebicka B., Wyska E., Grabowski T. (2007). Sevoflurane increases fade of neuromuscular response to TOF stimulation following rocuronium administration in children. A PK/PD analysis. Paediatr. Anaesth..

[18] Kalsotra S., Rice-Weimer J., Tobias J.D. (2023). Intraoperative electromyographic monitoring in children using a novel pediatric sensor. Saudi J. Anaesth..

[19] Kozák M., Szatmári K., Németh E., Fülesdi B., Nagy A., Tóth A., Boczán J. (2025). Function of the Normal Neuromuscular Junction in Young Children. Muscle Nerve.

